# Anthropometric, biochemical and clinical assessment of malnutrition among Egyptian children with chronic liver diseases: a single institutional cross-sectional study

**DOI:** 10.1186/s12876-019-1145-3

**Published:** 2019-12-21

**Authors:** Nehal El Koofy, Eman Mohamed Ibraheim Moawad, Mona Fahmy, Mona Anwar Mohamed, Hany Fathy Ahmed Mohamed, Ehab Mohamed Eid, Moushira Errfan Zaki, Rokaya Mohamed El-Sayed

**Affiliations:** 1 Department of Pediatrics, Faculty of Medicine, Cairo University, P.O.B. 126, Giza, Cairo, Egypt; 2 Academy of Scientific Research, Cairo, Egypt; 3 Medical Research Division, National Research Center, Cairo, Egypt; 4 Biological Anthropology, National Research Centre, Cairo, Egypt; 5 Institute of Postgraduate Childhood Studies, Ain Shams University, Cairo, Egypt

**Keywords:** Malnutrition, Children, Chronic liver diseases, Nutritional assessment, Anthropometry

## Abstract

**Background:**

Malnutrition is a common problem among children with chronic liver diseases (CLD). We aimed to assess the nutritional status of children with CLD and to correlate the anthropometric indices with the severity of liver disease, liver function tests, insulin growth factor-1 (IGF-1) and 25-hydroxy vitamin D (25- OH D).

**Methods:**

A total of 69 patients with CLD and 50 healthy controls (6 months − 6 years) were included in the study. Nutritional status was assessed by anthropometric indices expressed in standard deviation score (Z score), biochemical, hematological and clinical parameters.

**Results:**

We found 52.2% of CLD patients underweight by weight for age (W/A); 50.2% were stunted by height for age/ length for age (HAZ or LAZ); and 39% exhibited wasting by weight/height or (length) for age (W/HZ or W/LZ) z scores analysis. The mean values of z scores for all anthropometric parameters were significantly correlated with unconjugated and conjugated bilirubin and INR (*p < 0.05*), except HAZ or LAZ. Also, a significant correlation to albumin was found, except for W/HZ or (W/LZ) (*p = 0.157*). The z scores < − 2 SD based on W/ H versus arm indicators showed significant differences in MUAC, UAA and AMA (*p < 0.001*). We found no correlation between anthropometric z-scores and the mean IGF-1 and (25- OH D) values (*p > 0.05*). Malnutrition was directly correlated with the severity of hepatic dysfunction, particularly, Child-Pugh C cases. The mean IGF-1 and (25- OH D) values were significantly correlated with the severity of liver disease (*p < 0.001*).

**Conclusions:**

Our results identified anthropometric arm indicators and MUAC/A measurements as an effective applied methods for assessing nutritional status in CLD children. Moreover**,** Integrating comprehensive clinical assessment, anthropometric measurements and objective biochemical analyses is essential for evaluation, follow-up and management of CLD children with variable degree of malnutrition.

## Background

Malnutrition is a quiet problem in children with chronic liver disease (CLD) that has been implicated in short and long-term morbidity and mortality in both pre- and post-transplant periods [1, 2]. The prevalence varied according to the severity of underlying liver disease and the methods employed to assess nutritional status [3]. Patients may present with a wide range of clinical abnormalities including protein-energy malnutrition (PEM), linear growth-retardation, fat soluble vitamin deficiency and hepatic osteodystrophy [4, 5]. This complex of underlying nutritional derangement usually occurs prior to the clinical manifestations of hepatic insufficiency [4, 6].

In CLD, severe malnutrition, including skeletal muscle mass loss (sarcopenia), usually induces adverse outcomes, such as ascites, hepatic encephalopathy and lower survival rates among cirrhotic and liver transplant patients [2, 7].

There is no diagnostic “gold standard” for assessment of nutritional in those patients [5, 8]. In this context, a comprehensive nutritional analysis effectively engaging dietary history, subjective global assessment, anthropometry and biochemical parameters should be considered in all children with CLD [6]. Other tools such as hand grip strength, whole body dual-energy x-ray absorptiometry (DEXA) and tetrapolar bioelectrical impedance analysis (BIA) have emerged as newer modalities for nutritional assessment in hepatic patients [5, 9].

The present study aimed to assess the anthropometric parameters based on direct (conventional) and indirect arm measurements and correlate anthropometric nutritional status with the severity of liver disease, liver function tests (LFT), insulin growth factor-1 (IGF-1) and 25-hydroxy vitamin D (25- OH D) in children with CLD.

## Methods

This analytic, cross-sectional study was given to a convenience sample of children aged 6 months to 6 years with a clinical diagnosis of CLD emerging from various etiologies. Over a four month period between August to November 2015, we included sixty nine patients regularly attended the Pediatric Hepatology Unit, New children Hospital, a tertiary referral center for pediatric liver disease. Additionally, fifty, apparently healthy children, matched for age and sex, presented to the general pediatric outpatient clinic at the same hospital and during the same period were randomly selected as a control group. Patients were excluded if they had associated comorbidities e.g. renal and cardiac insufficiency, received a liver transplant and who were very ill e.g. tense ascites and massive edema. Children presented with acute liver cell failure without preexisting liver disease, those who had any metabolic or endocrinal diseases independently affecting nutritional status and whose caregivers didn’t consent to participate were also excluded.

In our study, cirrhosis was diagnosed histologically or defined by evidence of CLD with the presence of portal hypertension or complications of hepatic decompensation based on clinical, biochemical and radiological findings [10]. The severity of liver disease was assessed according to Child-Pugh score criteria [11]. Based on this scoring system, patients were classified into mild, moderate and severe disease. Scores > 15 were considered the cutoff point for liver disease severity.

### Study instruments

Nutritional status was evaluated based on anthropometric measurements and analytical parameters.

#### Anthropometric nutritional assessment

Three consecutive anthropometric measurements were taken by a single trained observer (one of the authors) for appraisal of nutritional status. To avoid possible errors, the average value was calculated immediately and used for the analysis. The basic anthropometric indicators used in the study were weight -for- age (W/A), height-for-age (H/A), head circumference-for-age (HC/A), and direct arm indicators including mid-upper arm circumference-for-age (MUAC/A) and triceps skinfold thickness-for-age (TSF/A) measured according to World Health Organization (WHO) reference techniques [12, 13].

Children < 2 years were weighed nude or in a clean, dry diaper using Seca infant scale. Children ≥2 years old were weighed privately and individually, barefooted with lightweight or no outer clothes and standing up in the center of the electronic scale platform without assistance. All weight measurements were recorded to the nearest 0.1 kg. Infantometer was used to measure length in children < 3 years, while standiometer with a movable block was used to measure height in those > 3 years [14].

A flexible, non-stretchable measuring tape was used over the most prominent part of the occiput and just above the supraorbital ridges to measure head circumference for all children ≤2 years old. The tape was also used to measure mid arm circumference while arm was held parallel to the body. The measurement was taken midway between the inferior border of the acromion process and the tip of the olecranon process. Triceps skinfold thickness was measured with Holtain skin fold caliper (Holtain Tanner/Whitehouse Skinfold Caliper, Holtain Limited, Pembrokeshire, UK) [15] at the previously marked midpoint used for MUAC. Length/height/A, HC/A and MUAC/A measurements of study participants were recorded to the nearest 0.1 cm.

Indirect arm indictors including, total upper arm area (UAA), arm muscle area (AMA) and arm fat area (AFA) were calculated with MUAC and total subcutaneous fat (TSF) measurements according to the formulas described by Jeliffe, Gurney and Frishancho [14, 16–18]. Results were expressed in a square millimeter as follows: AMA (cm^2^) = MUAC (cm) – [0.314 x TSF (cm) ^2^] /4 × 3.14; AFA (cm^2)^ = UAA (cm^2)^ – AMA (cm^2)^ and UAA (cm^2)^ = MAC^2^ **/**4 × 3.14.

Anthropometrical indicators were expressed in the form of standard deviation score (Z score), utilizing WHO Anthro (version 3.1) /2010 and WHO Anthro Plus/2007 software [13]. The cut off points of the − 2 z score were used to classify the nutritional status of the children into underweight, stunted and wasted child based on W/A, H/A and W/H Z scores (WAZ, HAZ and WHZ scores, respectively). Children who had Z score between - 1 and − 2 were considered to be at risk of malnutrition [12, 13].

#### Biochemical profile analysis

Blood samples collected from all patients were complete blood count (CBC) and LFT, including aspartate aminotransferase (AST), alanine aminotransferase (ALT), gamma glutamyl transferase (GGT), alkaline phosphatase (ALP), serum albumin, serum bilirubin (total and direct), prothrombin time (PT) and international normalized ratio (INR). All laboratory tests were performed according to the standard operating procedures in Biochemistry Laboratory of our hospital.

Insulin growth factor-1 was measured by enzyme linked radioimmunoassay (ELISA) after acid-ethanol extraction using kits from DIA source Immunoassays S. A, (Belgium) according to the manufacturer’s protocol. Plasma level of 25- OH D was measured in several batches using a radioimmunoassay with a method based on a high performance liquid chromatography (Incstar Corp, Stillwater, Minnesota, USA) [19].

All clinical and laboratory data were obtained at the same time of nutritional assessment. Maintenance and calibration guidelines to ensure the accuracy and reliability of childhood growth measurement and laboratory equipment were being followed.

### Statistical analysis

Data were statistically described in terms of mean ± standard deviation (± SD) or frequencies (number of cases) and percentages when appropriate. For categorical variables, absolute numbers and percentages will be calculated, together with their 95% confidence intervals. The comparison of means will be carried out using Student’s T test, the Mann–Whitney test, ANOVA test and Kruskall-Wallis test as appropriate. The association of qualitative variables will be carried out using Chi-square statistics. The correlations among quantitative variables were assessed using Pearson and Spearman’s correlation coefficient. *P* value < 0.05 was considered significant. All statistical calculations were done using computer program SPSS (Statistical Package for the Social Science; SPSS Inc., Chicago, IL) release 15 for Microsoft Windows (2006).

## Results

A total of 69 patients with CLD (Group I) and 50 healthy controls (Group II) were recruited during the study period. Table 1 highlights the basic demographic data and clinical features of the study participants. The mean age in CLD patients was 2.1 years (SD = 1.5), with significantly more boys participating than girls (52.2%, *p < 0.01*). The demographic variables, including age and gender were also comparable in the healthy control group (*p > 0.05*). Patients with biliary atresia (*n* = 39) comprised the largest group in the study (56.5%) followed by neonatal hepatitis (14.5%). The majority of patients had advanced liver disease with 35 cases (50.8%) of Child-Pugh B.

**Table 1:** Demographic and clinical characteristics of the study participants

Variable	Group I (Cases) *n* = 69 (%)	Group II (Controls) *n* = 50 (%)	P value
Gender			
Males	36 (52.2)	27 (54)	0.855
Females	33 (47.8)	23 (46)	
Age (years) [mean ± SD (range)	2.1 ± 1.5 (0.6–5)	2 ± 1.5 (0.5–5.8)	0.720
< 2 yr	0.8 ± 0.3 (0.6–1.9) (*n* = 48)	0.9 ± 0.4 (0.5–1.5) (*n* = 28)	0.841
2–6 y	2.5 ± 1.1 (2.1–5) (*n* = 21)	3.4 ± 1.2 (2–5.8) (*n* = 22)	0.584
Etiology of chronic liver disease		–	–
Biliary atresia	39 (56.5)		
Neonatal hepatitis	10 (14.5)		
PFIC	7 (10.1)		
Glycogen storage disease	5 (7.3)		
Allagile syndrome	4 (5.8)		
CHF Child-Pugh classification	4 (5.8)		
Chlild-Pugh A	17 (24.6)	–	–
Child-Pugh B	35 (50.8)		
Child-Pugh C	17 (24.6)		

Data are presented as mean ± standard deviation (SD), number (%) as appropriate. *CHF* congenital hepatic fibrosis; *PFIC* progressive familial intrahepatic cholestasis

**Fig. 1:**
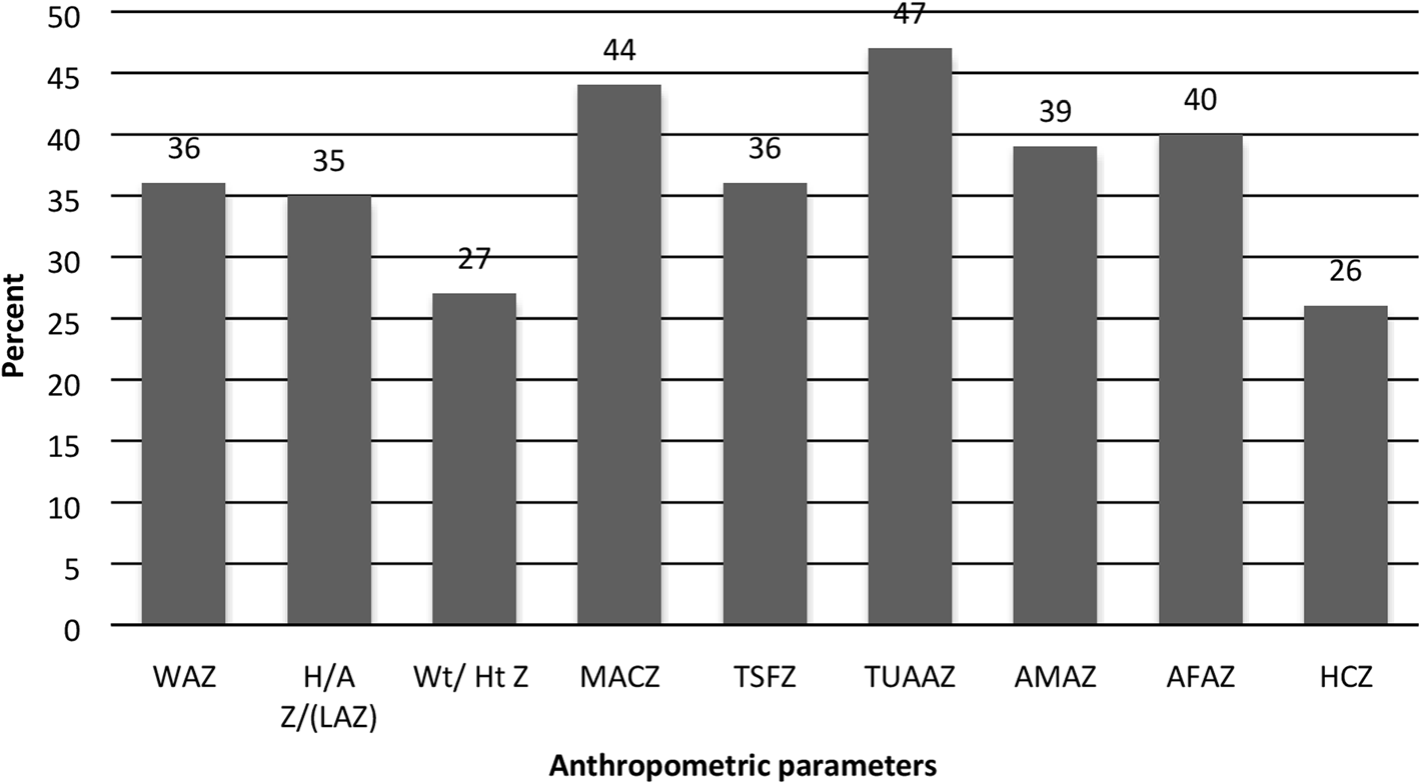
The percent distribution of anthropometric Z score below − 2 standard deviation (SD) in chronic liver disease children

The prevalence of malnutrition among the study cases is illustrated in Fig. 1. Based on WAZ, 52.2% were underweight, with the remaining 47.8% were at nutritional risk of being underweight. Approximately, half of the studied sample in Group I has been identified as being stunted upon HAZ or LAZ analysis.

There was a statistically significant difference between the studied groups as regard different measurements of the anthropometric parameters (*p < 0.001*) Table 2. Generally, the mean z-score of all anthropometric parameters is < zero, a finding denotes that all CLD patients have been nutritionally affected. Thirty-nine percent of the diseased children exhibited wasting (W/HZ or W/LZ < − 2SD), predominantly in the muscle portion with a mean value of MUACZ and AMAZ were − 3.9 (SD = 2.3) and − 2.9 (SD = 1.5), respectively (Table 2). Compared with other mean values, UAA has identified 68.1% (*n* = 47) of cases with z scores < − 2 SD. Head circumference for age in 52 patients aged < 2 years had z scores < − 2 SD in 50% of them (*n* = 26). Statistical comparison of cases with z scores < − 2 SD based on W/H (L) versus arm indicators showed significant differences in MUAC, UAA and AMA (*p < 0. 001*). This difference had no statistical significance when W/H (L) compared with TSF, AFA and HC/A (*p > 0.05*) within the same group. The majority of subjects in the healthy control group were well-nourished (92%); the remaining 4% were found to be at risk of malnutrition Table 2.

**Table 2:** Mean Z score values for the anthropometric parameters of the studied groups

Anthropometric indicators^*^	Group I (Cases) (n = 69)	Group II (Controls) (n = 50)
	Z score (mean ± standard deviation)	Z score (mean ± standard deviation)^*^
WAZ	−2.6 ± 1.5	0.01 ± 1.3^**^
HAZ or (LAZ)	− 2.2 ± 1.6	1.1 ± 1.5^**^
WT/ HT Z or (WtT/LZ)	−1.7 ± 1.5	0.9 ± 1.6^**^
MUCZ	− 3.9 ± 2.3	2.0 ± 1.7^**^
TSFZ	−2.2 ± 1.7	1.3 ± 0.8^**^
UAAZ	−3.6 ± 1.5	1.9 ± 1.3^**^
AMAZ	−2.9 ± 1.5	1.5 ± 1.1^**^
AFAZ	−2.4 ± 1.3	1.7 ± 0.9^**^
HCZ	− 2 ± 1.1	1.4 ± 0.8^**^

*WAZ* Weight for age z score, *HAZ* Height for age z score, *W/HZ* Weight/Height z score, *MUCZ* mid-upper arm circumference z score, *TSFZ* triceps skinfold z score, *UAAZ* upper arm area z score, *AMAZ* arm muscle area z score, *AFAZ* arm fat area z score, *HCZ* head circumference z score (only for children < 2 years)

^*^ All anthropometric indicators are calculated for age. ^**^highly significant with p (< 0.001)

Table 3 provides the results of mean values of biochemical and hematological parameters for the overall individuals studied. There were highly significant differences between CLD patients and healthy control groups in terms of LFT, IGF-1 and 25- OH D (*p < 0.001*). Based on hematologic analysis, only the mean hemoglobin values were significantly different between both groups (*p < 0.001*). Ninety-three percent of Group I (*n* = 64) had abnormal levels of AST, while the GGT levels exceeded the threshold values in 81% of cases (*n* = 56). Of 69 patients, 18 (26%) had PT three seconds more than control and 27 (39%) had INR > 1. Anemia was confirmed in 59.4% (*n* = 41) and 8% (n = 4) of Group I and II, respectively.

**Table 3:** Distribution of values of the biochemical and hematological parameters in the studied groups

Serum values (normal values)	Group I (Cases) (n = 69)	Group II (Controls) (n = 50)	P value
Conjugated Bilirubin (μmol /L) (< 3.4 μmol/L) [median, IQR]	70.1 (129.9) (1.7–205.2)	1.71 (0.34) (1.7–3.4)	< 0.001
Total Bilirubin (μmol /L) (< 21 μmol/L) [median, IQR]	111.5 (181.3) (1.7–388.3)	8.55 (3.42) (5.1–13.7)	< 0.001
AST (U/L) (< 1 year (15–60); 1–3 years (20–60); 4–6 years (15–50) [median, IQR]	117 (130.5) (19–831)	29.5 (16.25) (18–48)	< 0.001
ALT (U/L) (13–45) [median, IQR]	72 (96) (14–472)	23 (10) (10–44)	< 0.001
GGT (U/L) [< 1 year boy (5–65); < 1 year girl (5–35); 1–5 years (0–23)] [median, IQR]	261 (531.5) (12–2164)	13 (12.25) (5–32)	< 0.001
ALP (U/L) [< 2 years (150–40);2–10 years (100–320)] [median, IQR]	461 (437.5) (79–1925)	194 (77) (147–403)	< 0.001
Albumin (μmol/L) [4–6 months (2.8–5); 7–12 months (3.2–5.7); 13–24 months (1.9–5); 25–36 months (3.3–5.8); 3–5 years (2.9–5.8)] [mean ± SD]	3.4 ± 0.8 (1.6–5)	4.2 ± 0.7 (3–5.5)	< 0.001
Prothrombin time (seconds) [< 1 year [13 (10.1–15.9); 1–5 years (11 (10.6–11.4)] [mean ± SD]	15.3 ± 3.5 (13–32)	12.9 ± 0.5 (12–13.9)	< 0.001
IGF-1(nmol/L) (2.22–37.13) [median, IQR]	3.14 (1.38) (1.78–5.09)	5.22 (0.99) (4.31–6.93)	< 0.001
25 (OH) D (nmol/L) (42.43–134.7) [mean ± SD]	63 ± 13.25 (33.25–98.5)	59.75 ± 9 (64.75–152.5)	< 0.001
INR [1 (0.96–1.04)] [mean ± SD]	1.2 ± 0.4 (1–3.2)	1	< 0.001
Hemoglobin (g/dl) [6 months-2 years (12); 2–6 years (12.5)]	10.1 ± 0.15 (6.7–14.1)	11.9 ± 1.4 (9.6–14.5)	< 0.001
Total leucocytic count (× 10^3^/μL) [6 months-2 years (8.5 (5–15.5); 2–6 years (8.1 (4.5–13.5)] [mean ± SD]	11.5 ± 4.7 (3.5–25)	10.5 ± 4.1 (4.8–14)	0.229
Platelets (× 10^3^/μL) [mean ± SD] [6 months-6 years (150–350)]	342.3 ± 194.1 (15–964)	279.1 ± 135.5 (190–856)	0.050

*ALP* Alkaline phosphatase; *ALT* Alanine aminotransferase; *AST* Aspartate aminotransferase; *GGT* Gamma glutamyl transferase, *IGF-1* Insulin- like growth factor 1*, INR*: International normalized ratio; *IQR* interquartile range; *SD* standard deviation; 25 (OH) D: 25- hydroxyvitamin D,

Table 4 shows the correlation of biochemical and hematological parameters with z scores of anthropometric indicators in Group I. Overall, HCZ, TSFZ and AFAZ had no significant correlation with all LFT measured in the study (*p > 0.05*). In terms of liver enzymes, all of the derived arm indicators including UAAZ, AMAZ and AFAZ had no significant correlation with AST, ALT and ALP (*p > 0.05*) except GGT. The mean values of z scores for all anthropometric parameters were significantly correlated with conjugated bilirubin and INR except HAZ or LAZ (*p = 0.213 and 0.678, respectively*). The correlation between albumin and participants’ anthropometric indicators were positively significant except for W/HZ or W/LZ (*p = 0.157*). Likewise, hemoglobin was significantly correlated with anthropometric measurements except for WAZ, HAZ or LAZ and HCZ.

**Table 4:** Correlation of biochemical and hematological parameters with anthropometric indicators in chronic liver disease patients

	Anthropometric Indicatorsǂ							
	WAZ	HAZ/ LAZ	WT/Ht (WT/LZ)	MUCZ	TSFZ	UAAZ	AMAZ	AFAZ
CB	−0.459	− 0.152	− 0.367	− 0.537	−0.469	− 0.530	−0.496	− 0.523
	< 0.001*	0.213	0.002*	< 0.001*	< 0.001*	< 0.001*	< 0.001*	< 0.001*
UCB	−0.478	−0.237	− 0.339	− 0.452	−0.421	− 0.423	−0.357	−.449
	< 0.001*	0.050*	0.004*	< 0.001*	< 0.001*	< 0.001*	0.003*	< 0.001*
Albumin	0.373	0.302	0.172	0.474	0.412	0.472	0.414	.363
	0.002*	0.012*	0.157	< 0.001*	< 0.001*	< 0.001*	< 0.001*	0.002*
INR	−0.302	−0.051	−0.270	− 0.311	− 0.316	− 0.305	−0.247	−.305
	0.012*	0.678	0.025*	0.009*	.008*	0.011*	0.023*	0.011*
ALT	0.215	0.267	0.128	0.122	−0.002	0.071	0.080	0.041
	0.075	0.026*	0.295	0.316	0.986	0.561	0.512	0.736
GGT	0.131	−0.073	−0.047	0.262	−0.059	−0.259	− 0.318	−0.182
	0.284	0.553	0.702	0.030*	0.632	0.031*	0.008*	0.135
Hemoglobin	0.2	0.05	0.29	0.47	0.479	0.436	0.068	0.425
	0.07	0.7	0.04*	< 0.001*	< 0.001*	0.002*	0.006*	0.002*
TLC	−0.310	−0.063	−0.397	−0.290	−0.379	− 0.402	−0.087	− 0.1391
	0.028*	0.664	0.004*	0.041*	0.007*	0.004*	0.551	0.341

*AFAZ* arm fat area z score; *AMAZ* arm muscle area z score; *ALP* Alkaline phosphatase; *ALT* Alanine aminotransferase; *AST* Aspartate aminotransferase; *CB* conjugated bilirubin; *GGT* gamma glutamyl transferase; *HAZ* Height for age z score; *HCZ* head circumference z score (only for children < 2 years); *IGF-1* Insulin Growth Factor-1; *INR* international normalized unit; *MUCZ* mid-upper arm circumference z score; *R* Correlation coefficient; *TLC* Total leucocytic count; *TSFZ* triceps skinfold z score; *WAZ* Weight for age z score; *W/HZ* Weight/Height z score; *UAAZ* upper arm area z score; *UCB* unconjugated bilirubin; *25(OH)D* 25-hydroxy vitamin D. P value is significant < 0.05; highly significant at < 0.001. ^ǂ^ All anthropometric indicators are calculated for age

Though 37.7% (*n* = 26) and 24.6% (*n* = 17) of the diseased children had abnormal IGF-1 and 25 - OH D values, respectively, yet they had no correlation to anthropometric z-scores of the CLD patients. Also, there was no statistically significant difference in the mean IGF-1 and (25- OH D) values of patients with anthropometric z –scores < − 2 SD compared to those with a mean > − 2 SD.

Table 5 illustrates the mean nutritional parameters of the CLD participants, IGF-1 and 25 (OH) D in relation to Child-Pugh grades. Malnutrition was directly correlated to the severity of hepatic dysfunction with a substantial number of Child-Pugh C cases being severely malnourished (Z score < − 2 SD). For example, the mean values of WAZ, HAZ or LAZ and W/HZ or W/LZ significantly demonstrated all aspects of malnutrition in terms of underweight, stunting and wasting more in Child-Pugh C children than in A (*p < 0.001, 0.05 and 0.05*, respectively). Among all the anthropometric indicators, MUACZ was the only parameter that had a mean value < − 5 SD reported in Child-Pugh C patients. A strong correlation was verified between IGF-1 as well as (25- OH D) mean values and Child-Pugh classes (*p < 0.004, p < 0.001,* respectively) Table 5.

**Table 5:** Mean anthropometric parameter Z scores, Insulin Growth Factor-1 (IGF-1) and 25-hydroxy vitamin D in relation to Child Pugh Classes in chronic liver disease patients

Anthropometric^ǂ^ Indicators	Mean ± SD of anthropometric indicators Z scores			P value
	Child Pugh A (n = 17)	Child Pugh B (n = 35)	Child Pugh C (n = 17)	
WAZ	−1.04 ± 0.8 ^ab^	−2.5 ± 1.4 ^b^	− 3.3 ± 1.4 ^a^	< 0.001*
HAZ or (LAZ)	− 1.33 ± 1.6 ^a^	− 2.3 ± 1.5 ^b^	−2.7 ± 1.5 ^a^	< 0.05*
W/HZ or (W/LZ)	− 0.49 ± 1.4 ^a^	−1.5 ± 1.6 ^b^	−1.8 ± 1.3 ^a^	< 0.05*
MUACZ	−1.4 ± 0.96 ^a^	−3.5 ± 2.2 ^a^	−5.3 ± 1.9 ^a^	< 0.00*
TSFZ	−0.73 ± 1.1 ^ab^	−1.6 ± 1.6 ^b^	− 3.6 ± 1.5 ^a^	< 0.00*
UAAZ	− 1.8 ± 0.9 ^a^	−3.1 ± 1.5 ^a^	−4.4 ± 1.3 ^a^	< 0.001*
AMAZ	−1.3 ± 0.6 ^a^	− 2.6 ± 1.5 ^a^	− 3.6 ± 1.4 ^a^	< 0.001*
AFAZ	−1.4 ± 0.8 ^a^	−1.9 ± 1.3 ^b^	−3.3 ± 0.9 ^ab^	< 0.001*
HCZ	− 1.2 ± 0.9 ^ab^	− 2.3 ± 1.2 ^b^	− 2.2 ± 1.1 ^a^	< 0.05*
IGF-1* (ng/ml)	28.1 ± 6.2 (17–28)	25.2 ± 4.7 (19–38.6)	22.3 ± 3.6 (15–26)	< 0.004**
25-hydroxy vitamin D* (ng/ml)	28.6 ± 5.7 (17.3–37)	23.4 ± 5.8 (13.3–33.5)	19.7 ± 4 (15.2–26.4)	< 0.001**

*AFAZ* arm fat area z score; *AMAZ* arm muscle area z score; *HAZ* Height for age z score; *HCZ* head circumference z score (only for children < 2 years); *MUCZ* mid-upper arm circumference z score; *TSFZ* triceps skinfold z score; *WAZ* Weight for age z score; *W/HZ* Weight/Height z score; *UAAZ* upper arm area z score.* P value is significant < 0.05; highly significant at < 0.001. a,b,c Within anthropometric parameter for each group, mean values that share the same superscript letter are statistically significantly different from each other. ^ǂ^ All anthropometric indicators are calculated for age

## Discussion

Anthropometric measurements are considered the most practical objective indices utilized for nutritional assessment in CLD [20]. Consistent with other studies [4, 21], more than half of the diseased children exhibited a range of nutritional abnormalities. Conventional anthropometric measurements such as W/H or W/L revealed wasting in approximately 40% of studied cases. Inadequate nutrient intake linked to anorexia, metabolic derangement, recurrent cholangitis and prolonged hospitalization are factors associated with malnutrition in CLD patients [22].

In the current study, direct and indirect arm indicators in the form of MUAC, UAA and UAMA have significantly increased the detection of malnourished cases by 1.6-, 1.7- and 1.5-fold, respectively (*p < 0.001*). These findings are consistent with other studies from developing countries in Africa [23] and South America [6, 8, 24]. This might be attributable to the fact that MUAC and UAA incorporate muscle tissue and fat stores [4, 25] that, respectively, undergo continuous accelerated loss and depletion in such patients [1, 26].

A fewer number of malnourished diseased children (*n* = 36) were diagnosed when TSF measurement was employed. Although these results were consistent with previous reports [4, 8], however, several studies have shown higher rates of malnutrition based on TSF measurement [26, 27]. This difference may be partially explained by the different approaches adopted by other researchers who obtained muscle adipose index (from arm indicators) to diagnose nutritional inadequacy. This emphasizes the importance of combining arm muscle indicators and TSF for the assessment of protein portion and fat content of CLD patients [28].

Alberino et al hypothesized that assessment of nutritional status could enhance the prognostic accuracy of Child-Pugh classification [29]. Similar to other studies [21, 30], we found that Child-Pugh C patients had a significantly lower anthropometric arm measurements compared to Child-Pugh B (*p* < 0.001), a finding indicates the ability of anthropometric upper arm indicators to differentiate nutritional status across classes of decompensated cirrhotic patients. Although AMA detected a greater frequency of malnutrition (77.1%) relative to AFA (60%) in Child class B, the situation was reversed for Child class C children whose AMA and AFA measurements demonstrated malnutrition in 94.1 and 82.4% of patients, respectively. These findings indirectly reflect sarcopenia that worsens with progression of the liver disease. This could be related to metabolic alteration associated with the severity of liver disease with lipids used as a preferential metabolic substrate for energy production [31].

Several previous studies [4] have found that conjugated bilirubin is negatively correlated with anthropometric parameters z-scores, a finding corroborated in our study. Although, serum albumin has low specificity and sensitivity as a nutritional index compared to pre-albumin, particularly in patients with CLD [32], yet, we found a significant positive correlation of albumin with all anthropometric z-scores, except W/H or W/L and H/C. This was in agreement with a study by Hurtado et al. conducted with LFT as independent variables and anthropometric indicators as dependent variables. The study showed that the nutritional status of children with CLD could be predicted by the biochemical liver indicators, predominantly with conjugated bilirubin and serum albumin [4]. Two other studies have identified increased unconjugated bilirubin, decreased serum albumin along with prolonged INR > 1 as factors associated with higher rates of intensive care unit admission and mortality [33, 34]. In the present study, ALP was identified as the most altered LFT that correlated negatively with all anthropometric parameters except W/A, although, this correlation was not statistically significant (*P > 0.05*). Furthermore, we found a significant negative correlation between GGT and arm indicators namely MUAC, UAA and AMA. Although these results share a number of similarities with Hurtado et al*,* however, there is concern over the prognostic value of those enzymes (ALP and GGT) which are not associated with hepatic synthesis [35].

Anemia of diverse etiology is a common manifestation associated with CLD [36]. We found a strong positive correlation between hemoglobin levels and most anthropometric parameters. Nevertheless, a stronger correlation is established with disease severity than with a degree of malnutrition in such patients [36]. We also observed a positive correlation between total leucocytic count (TLC) and most of anthropometric measurements. However, some factors such as metabolic stress and immunosuppression may affect reliability of TLC as a nutritional indicator in CLD patients [37].

Serum level of IGF-1 has been constantly found to correlate with anthropometric indices [38, 39]. Overall, the mean serum IGF-1 value was significantly lower in cases compared to controls (*p < 0.001*). We also found a significant alteration (*p < 0.004*) of the mean IGF-1 level with the severity of liver disease, being the lowest in Child-Pugh C. Colakoglu et al [40] found IGF-1 and Insulin-like growth factor-binding protein 3 levels were significantly lower in 42 cirrhotic patients compared to non-cirrhotic ones. A previous Egyptian study found children with CLD of various etiologies had a significant lower mean IGF-1 relative to healthy controls (*p < 0.05*). Conversely, they showed that serum IGF-1 mean levels were directly associated with the grade of liver damage with the highest value recorded in class C [41]. This difference might be attributable to sampling bias because a small proportion of their total sample (*n* = 50) was classified as Child-Pugh C (*n* = 9, 4.8%). Also, they investigated CLD secondary to viral hepatitis (B and C) and Bilharziasis, causes not involved in our study.

We couldn’t establish a significant relation between mean IGF-1 level and the anthropometric z-score (*p > 0.05*). Similarly, previous studies evaluating the relation between IGF-1 level and nutritional status among critically ill children have yielded conflicting results [42, 43]. These findings indicate that there is a considerable need for further research on IGF-1 in relation to different nutritional indices in CLD patients.

Consistent with other studies [44], we found a significant difference in mean (25- OH D) levels between cases and controls (*p < 0.001*). In this context, malabsorption, inadequate dietary intake and lack of sun exposure may be contributing factors to vitamin D deficiency (defined as 25- OH D concentration < 20 ng/ml in the pediatric population) in such patients [8, 45, 46]. A study in Brazil reported direct correlation between most of anthropometric indices and serum levels of vitamin A and E. Nonetheless, they couldn’t verify the relation between those indices and vitamin D, a finding reproduced in our study.

To the best of our knowledge, no Egyptian studies comprehensively investigated the nutritional status of CLD in pediatric age group incorporating clinical, anthropometric and biochemical analyses. This study may, therefore, substantiate the necessity to develop robust assessment strategies that can either help early screening of malnutrition or create distinctive tools for prognosis of morbidity and mortality accompanying malnutrition. Our data may be useful in selection of recipients for liver transplantation which could be the most important factor in determining long-term survival. Also, our findings suggest that preventive measures of malnutrition in CLD children should prioritize infant age group. There were some limitations such as a relatively small sample size and certain limitation for selection determined by eligibility criteria that allowed patients to be enrolled regardless of the stage of their liver disease.

## Conclusion

Our results identified anthropometric arm indicators and MUAC/A measurement as an effective applied methods for assessing nutritional status in CLD children. Integrating comprehensive clinical assessment, anthropometric measurements and objective biochemical analyses such as albumin and conjugated bilirubin is essential for evaluation, follow-up and management of CLD children with variable degree of malnutrition.

## Data Availability

The datasets generated and/or analysed during the current study are not publicly available to maintain participant confidentiality, but are available from the corresponding author on reasonable request.

## References

[1] Patton HM (2012). Nutritional assessment of patients with chronic liver disease. Gastroenterol Hepatol (N Y).

[2] Gz P, Oliveira K, Soldera J (2014). Biochemical nutritional profile of liver cirrhosis patients with hepatocellular carcinoma. Arq Gastroenterolo.

[3] Amin HM, Abdel Samie RM, Hamed FS (2016). Assessment of Nutritional Status of Patients with Chronic Hepatitis C and HCV-Related Cirrhosis in the Compensated Stage. J Intern Med.

[4] Hurtado-López EF, Larrosa-Haro A, Vásquez-Garibay EM, Macías-Rosales R, Troyo-Sanromán R, Bojórquez-Ramos MC (2007). Liver function test results predict nutritional status evaluated by arm anthropometric indicators. J Pediatr Gastroenterol Nutr.

[5] Nunes G, Santos CA, Barosa R, Fonseca C, Barata AT, Fonseca J (2017). Outcome and nutritional assessment of chronic liver disease patients using anthropometry and subjective global assessment. Arq Gastroenterol.

[6] Zamberlan P, Leone C, Tannuri U, Carvalho WB, Delgado AF (2012). Nutritional risk and anthropometric evaluation in pediatric liver transplantation. Clinics..

[7] Mandato Claudia, Di Nuzzi Antonella, Vajro Pietro (2017). Nutrition and Liver Disease. Nutrients.

[8] Henkel AS, Buchman AL (2006). Nutritional support in patients with chronic liver disease. Nat Clin Pract Gastroenterol Hepatol.

[9] European Association for the Study of the Liver (2019). EASL clinical practice guidelines on nutrition in chronic liver disease. J Hepatol.

[10] Schuppan D, Afdhal NH (2008). Liver cirrhosis. Lancet.

[11] Pugh RN, Murray-Lyon IM, Dawson JL, Pietroni MC, Williams R (1973). Transection of the oesophagus for bleeding oesophageal varices. Br J of Sur.

[12] World Health Organization. WHO child growth standards: methods and development: length/height-for-age, weight-for-age, weight-for-length, weight-for-height and body mass index-for-age. Geneva: World Health Organization. 2006.

[13] World Health Organization, World Health Organization. Nutrition for Health. WHO child growth standards: head circumference-for-age, arm circumference-for-age, triceps skinfold-for-age and subscapular skinfold-for-age: methods and development. World Health Organization; 2007.

[14] Frisancho AR. Anthropometric standards for the assessment of growth and nutritional status: University of Michigan Press; 1990.

[15] Center for Disease Control and Prevention. National Health and Nutrition Examination Survey (NHAMES) Anthropometry Procedures Manual. Available online: http://www.cdc.gov/nchs/data/ nhanes/nhanes_07_08/manual_an.pdf (accessed on 7/03/2015).

[16] Jelliffe DB (1963). The incidence of protein-calorie malnutrition of early childhood. Am J Public Health.

[17] Gurney JM, Jelliffe DB (1973). Arm anthropometry in nutritional assessment: monogram for rapid calculation of muscle circumference and cross-sectional muscle and fat areas. Am J Clin Nutr.

[18] Sann L, Durand M, Picard J, Lasne Y, Bethenod M (1988). Arm fat and muscle areas in infancy. Arch Dis Child.

[19] Hollis BW, Kamerud JQ, Selvaag SR, Lorenz JD, Napoli JL (1993). Determination of vitamin D status by radioimmunoassay with an 125I-labeled tracer. Clin Chem.

[20] Matos C, Porayko MK, Francisco-Ziller N, DiCecco S (2002). Nutrition and chronic liver disease. J Clin Gastroenterol.

[21] Tai ML, Goh KL, Mohd-Taib SH, Rampal S, Mahadeva S (2010). Anthropometric, biochemical and clinical assessment of malnutrition in Malaysian patients with advanced cirrhosis. Nutr J.

[22] Bavdekar A, Bhave S, Pandit A (2002). Nutrition management in chronic liver disease. Indian J Pediatr.

[23] Israëls Trijn, Chirambo Chawanangwa, Caron Huib N., Molyneux Elizabeth M. (2008). Nutritional status at admission of children with cancer in Malawi. Pediatric Blood & Cancer.

[24] Nel ED, Terblanche AJ (2015). Nutritional support of children with chronic liver disease. SAMJ.

[25] Silva FV, Ferri PM, Queiroz TC (2016). Nutritional evaluation of children with chronic cholestatic disease. J Pediatr.

[26] Wanke C, Polsky B, Kotler D (2002). Guidelines for using body composition measurement in patients with human immunodeficiency virus infection. AIDS Patient Care STDs.

[27] García-Rodríguez MT, del Carmen P-VM, López-Calviño B (2015). Assessment of nutritional status and health-related quality of life before and after liver transplantation. BMC Gastroenterol.

[28] Sokol RJ, Stall C (1990). Anthropometric evaluation of children with chronic liver disease. Am J Clin Nutr.

[29] Alberino F, Gatta A, Amodio P (2001). Nutrition and survival in patients with liver cirrhosis. Nutrition.

[30] Santetti D, de Albuquerque Wilasco MI, Dornelles CT (2015). Serum proinflammatory cytokines and nutritional status in pediatric chronic liver disease. WJG..

[31] Vieira PM, De-Souza DA, Oliveira LC (2013). Nutritional assessment in hepatic cirrhosis; clinical, anthropometric, biochemical and hematological parameters. Nutr Hosp.

[32] Jensen GL (2006). Inflammation as the key interface of the medical and nutrition universes: a provocative examination of the future of clinical nutrition and medicine. J Parenter Enteral Nutr.

[33] Malatack JJ, Schaid DJ, Urbach AH (1987). Choosing a pediatric recipient for orthotopic liver transplantation. J Pediatr.

[34] McDiarmid SV, Anand R, Lindblad AS (2002). Development of a pediatric end-stage liver disease score to predict poor outcome in children awaiting liver transplantation1. Transplantation.

[35] Hsu EK, Murray KF, Suchy F (2014). Cirrhosis and chronic liver failure. Liver disease in children.

[36] Mattar RH, Azevedo RA, Speridião PG, Fagundes Neto U, Morais MB (2005). Nutritional status and intestinal iron absorption in children with chronic hepatic disease with and without cholestasis. J Pediatr.

[37] Bharadwaj S, Ginoya S, Tandon P, Gohel TD, Guirguis J, Vallabh H, Jevenn A, Hanouneh I. Malnutrition: laboratory markers vs nutritional assessment. Gastroenterol Rep. 2016;4(4):272-80. 10.1093/gastro/gow013 PMC5193064 27174435

[38] Campillo B, Paillaud E, Bories PN, Noel M, Porquet D, Le Parco JC (2000). Serum levels of insulin-like growth factor-1 in the three months following surgery for a hip fracture in elderly: relationship with nutritional status and inflammatory reaction. Clin Nutr.

[39] Donahue SP, Phillips LS (1989). Response of IGF-1 to nutritional support in malnourished hospital patients: a possible indicator of short-term changes in nutritional status. Am J Clin Nutr.

[40] Colako O, Taskiran B, Colako G (2007). Serum insulin like growth factor-1 (IGF-1) and insulin like growth factor binding protein-3 (IGFBP-3) levels in liver cirrhosis. Turk J Gastroenterol.

[41] Mahdy KA, Ahmed HH, Mannaa F, Abdel-Shaheed A (2007). Clinical benefits of biochemical markers of bone turnover in Egyptian children with chronic liver diseases. World J Gastroenterol.

[42] Leite HP, Fisberg M, Vieira JG, De Carvalho WB, Chwals WJ (2001). The role of insulin-like growth factor I, growth hormone, and plasma proteins in surgical outcome of children with congenital heart disease. Pediatr Crit Care Med.

[43] Balcells J, Moreno A, Audí L, Roqueta J, Iglesias J, Carrascosa A (2001). Growth hormone/insulin-like growth factors axis in children undergoing cardiac surgery. Crit Care Med.

[44] Silveira TR, Salzano FM, Donaldson PT, Mieli-Vergani G, Howard ER, Mowat AP (1993). Association between HLA and extrahepatic biliary atresia. J Pediatr Gastroenterol Nutr.

[45] Misra M, Pacaud D, Petryk A, Collett-Solberg PF, Kappy M (2008). Vitamin D deficiency in children and its management: review of current knowledge and recommendations. Pediatrics.

[46] Ross AC, Taylor CL, Yaktine AL, Del Valle HB, editors. Dietary reference intakes for calcium and vitamin D. National Academies Press; 2011. 21796828

